# Disease activity trajectories in paediatric lupus and associations with socioeconomic factors and patient-reported pain

**DOI:** 10.1136/lupus-2025-001521

**Published:** 2025-08-14

**Authors:** Siobhan Case, C Larry Hill, Peter Shrader, Anne Dennos, Thomas Phillips, Laura Eve Schanberg, Emily von Scheven, Kamil Barbour, Andrea M Knight, Aimee Hersh, MaryBeth Son, for the CARRA Registry Investigators, R Aamir

**Affiliations:** 1 Brigham and Women’s Hospital, Boston, Massachusetts, USA; 2 Boston Children’s Hospital, Boston, Massachusetts, USA; 3 Department of Pediatrics, Harvard Medical School, Boston, Massachusetts, USA; 4 Duke University Duke Clinical Research Institute, Durham, North Carolina, USA; 5 Pediatrics, Duke University, Durham, North Carolina, USA; 6 Pediatrics, UCSF School of Medicine, San Francisco, California, USA; 7 National Institutes of Health, Bethesda, Maryland, USA; 8 Rheumatology, Hospital for Sick Children, Toronto, Ontario, Canada; 9 University of Utah Health, Salt Lake City, Utah, USA

**Keywords:** Lupus Erythematosus, Systemic, Patient Reported Outcome Measures, Autoimmune Diseases, Pain

## Abstract

**Objective:**

Using data from participants with paediatric SLE (pSLE) in the Childhood Arthritis and Rheumatology Research Alliance Registry, we aimed to: (1) describe 2-year disease activity trajectories, measured by the SLE Disease Activity Index 2000 (SLEDAI 2K); (2) identify characteristics associated with each trajectory and (3) assess achievement of lupus low disease activity state (LLDAS) and associated baseline characteristics.

**Methods:**

Participants were diagnosed with pSLE within 12 months of baseline visit. Baseline sociodemographic, clinical and treatment characteristics were included in latent trajectory analyses. Associations between patient characteristics with trajectory groups and LLDAS were analysed with multinomial generalised logistic regression modelling.

**Results:**

1002 patients were screened; 553 were included for SLEDAI 2K and 269 for LLDAS analyses. SLEDAI 2K trajectories included (T1) low and stable, (T2) high and decreasing, (T3) intermediate and stable. In multinomial generalised logistic regression, baseline SLEDAI 2K score and insurance type were significantly associated with trajectories. 51% (136/269) of patients achieved LLDAS at least once in 24 months as compared with 17% (47/269) at first assessment. LLDAS attainment at both time points was predicted by lower pain interference scores; LLDAS attainment over 24 months was also associated with baseline American College of Rheumatology classification criteria, rituximab use at baseline and highest completed level of parent/guardian education.

**Conclusions:**

Disease activity trajectories in a pSLE cohort were predicted by baseline SLEDAI 2K and insurance. Only half of the patients achieved LLDAS during the 2-year study period, which was predicted by baseline characteristics including pain interference. The relationship between disease activity and socioeconomic factors and pain warrants further investigation to identify modifiable factors to reduce pSLE disease activity.

**WHAT IS ALREADY KNOWN ON THIS TOPIC**

- While wide variation in the clinical course of paediatric SLE (pSLE) is clinically recognised, it has been challenging to characterise trajectories of disease activity across a diverse cohort.

**WHAT THIS STUDY ADDS**

- This study in a diverse cohort of 553 patients with pSLE identified three 2-year disease activity trajectories (as measured by SLE Disease Activity Index 2000 (SLEDAI 2K)); the trajectory with higher disease activity over time was associated with higher baseline SLEDAI 2K scores and lack of health insurance. Lower rates of lupus low disease activity state achievement in 269 patients with pSLE were associated with higher levels of pain interference at baseline.

**HOW THIS STUDY MIGHT AFFECT RESEARCH, PRACTICE OR POLICY**

- Variables related to socioeconomic status and patient-reported pain outcomes appear to influence the course of illness in pSLE. Patients with pSLE with high SLEDAI 2K scores at baseline should be closely monitored for persistent or worsening disease activity. The role of pain in patients with pSLE warrants further investigation.

##  Introduction

The disease course of paediatric SLE (pSLE) is highly variable, with some patients following a chronic but manageable course while others experience life-threatening flares with significant morbidity and disability. Compared with adult-onset SLE, pSLE is associated with more severe manifestations including more frequent neurological and renal involvement, higher mortality,^1^ increased exposure to immunosuppression and higher accrual of disease damage.^2^ Compounding these issues, there are many documented disparities in pSLE as children identifying with certain racial and ethnic groups are more likely to develop pSLE and experience severe manifestations.^3^,^5^ However, risk factors for severe disease trajectories are not fully understood, leaving questions as to how to identify and treat patients with pSLE at highest risk of severe disease with the goal of improving health outcomes.

Trajectories of disease activity in pSLE have been described for an inception cohort of patients in Ontario, Canada seen between 1985 and 2011.^6^ Disease activity trajectories were associated with disease damage trajectories, as well as demographic and clinical characteristics such as self-identified ethnicity. The same cohort was used to study the trajectory of disease damage, revealing a steady accumulation of damage during adulthood.^7^ Predictors of damage trajectories also included self-reported ethnicity, as well as earlier time periods of diagnosis (1985–1990 vs 1991–2000 vs 2001–2011), treatment with cyclophosphamide and higher doses of prednisone. However, these studies did not include measures of socioeconomic status (SES) or patient-reported outcomes (PRO) and spanned an era prior to the use of rituximab and other biological treatments in SLE. Additionally, another study of patients with childhood-onset SLE in the Childhood Arthritis and Rheumatology Research Alliance (CARRA) Registry found that PRO including pain interference did not clearly predict active versus inactive disease but was likely limited by the small sample size (n=58).^8^ It is not clear how these associations might hold in a larger population with contemporary treatment standards.

In this study of participants with pSLE enrolled in the CARRA Registry, we describe disease trajectories in a large, diverse cohort of patients across North America and use ZIP code level measures of SES. The study aimed to (1) describe 2-year trajectories of disease activity as measured by the SLE Disease Activity Index 2000 (SLEDAI 2K)^9^; (2) identify key baseline (ie, at enrolment) patient characteristics associated with different trajectories; and (3) describe the association between baseline characteristics and presence of lupus low disease activity state (LLDAS)^10^ at the first assessment (determination of LLDAS response requires data from two visits) as well as LLDAS attainment at any point in the first 24 months of Registry enrolment.

## Patients and methods

### Study design, data source and patient involvement

The CARRA Registry enrols patients from 71 sites across North America. For this analysis, data were included from March 2017 through August 2022.^11^ Patients and their families were not directly involved in setting the research question, study design or analysis.

### Subjects

Participants in the CARRA Registry were eligible for the current analysis if they had a diagnosis of pSLE as defined by the Systemic Lupus International Collaborating Clinics (SLICC) criteria,^12 13^ had no diagnosis of a secondary rheumatological condition other than antiphospholipid antibody syndrome or macrophage activation syndrome, and were enrolled in the Registry within 12 months of diagnosis. Exclusion criteria included an insufficient number of visits within the first 24 months of enrolment to calculate trajectories for the disease activity measure (less than two Registry visits for the SLEDAI 2K, or less than three Registry visits for the LLDAS).

### Data collection

Baseline demographics included sex, self-reported race and ethnicity, highest parent/guardian education level, household income, health insurance status and 9-digit ZIP code which was used to calculate the national neighbourhood Area Deprivation Index (ADI) using US census bureau data from 2010.^14^ Higher ADI signifies more deprivation and lower SES (range 1–100). Insurance was categorised as none, private, government (ie, Medicare, Medicaid, state and/or military insurances) or other.

Baseline clinical characteristics included age at diagnosis, age at symptom onset, days from symptom onset to diagnosis, days from diagnosis to baseline visit, immunosuppressive medications (including glucocorticoids) prior to or at baseline visit, days from diagnosis to first immunosuppressive medication, number of American College of Rheumatology (ACR) and SLICC classification criteria,^12 13^ SLEDAI 2K score (range 0–105),^9^ SLICC/ACR Damage Index (SDI) (range 0–47),^15^ attainment of LLDAS as a binary measure (LLDAS score computed using 0 for any missing components, attainment scored as yes/no),^10^ presence of lupus nephritis (as determined by physician report), physician global assessment, PRO comprising patient/parent global assessment and Patient-Reported Outcomes Measurement Information System (PROMIS) measures of pain intensity (how much pain a patient experiences, range 0–10) and interference (how pain interferes with well-being and daily activities, range 0–32), and the paediatric global health survey.^16^,^18^ Lower scores on the pain-related PROMIS measures indicate lower pain intensity/pain interference and higher scores on the paediatric global health survey indicate better overall health and quality of life. Oral glucocorticoid use was categorised into low/intermediate dose and high dose, with high dose defined as ≥20 mg a day.

Data for SLEDAI 2K and LLDAS were collected from outpatient scheduled (every 6 months) or unscheduled (for medications changes) visits and were assigned to a visit allowing for a 3-month window around each 6-month interval. If more than one visit occurred in the same window, the date collected closest to the 6-month interval was selected.

### Statistical analysis

We compared baseline characteristics between participants who were included versus excluded, with χ² tests for categorical and Wilcoxon rank sum tests (non-parametric statistical methods) for continuous variables, respectively. A p value of<0.05 was considered significant.

Latent trajectory analysis was performed on the SLEDAI 2K scores using SAS procedure Proc Traj (SAS Institute, Cary, North Carolina, USA). The maximum likelihood method was used based on the SLEDAI 2K scores. No covariates were used in the trajectory modelling. All potential models were performed that had 2–5 trajectories, with any possible combination of constant, linear or quadratic terms. Within each model, the posterior probability of being assigned to each trajectory was calculated. Patients were then assigned the trajectory in which they had the highest probability. The Bayesian information criterion (BIC), Akaike information criterion (AIC) and average of the posterior probability of all subjects assigned to each trajectory were calculated for each model. Models were assessed based on BIC and AIC, as well as posterior probability >0.75 and n>30 for all trajectory groups. The clinical relevance of each model was assessed and one final model was selected.

Baseline characteristics among (1) each SLEDAI 2K trajectory; (2) LLDAS attainment at first assessment (ie, at the second registry visit) or (3) ever achieving LLDAS within 24 months were described with summary statistics. Trajectory groups were compared using the Wilcoxon rank sum test for continuous variables and χ² test for categorical variables at a significance level of <0.05.

Three separate multinomial generalised logistic regression models examined associations between baseline patient characteristics and (1) SLEDAI 2K trajectory groups, (2) LLDAS at first assessment and (3) ever achieving LLDAS within 24 months. The reference category for the SLEDAI 2K trajectory group was T1 (low baseline score with stable trajectory) and for LLDAS was not being in a low disease state. In the case of missing, not answered or unknown information, the data were imputed using a multiple imputation process with 20 imputation models, obtained using Markov Chain Monte Carlo simulation or regression methods. Results of the multiple imputation were analysed for variability between imputations. Less than 10% missingness was reported in most variables, so there was low variability between the multiple imputations. We used forward variable selection; variables needed to have a p value of ≤0.05 to remain in the model. Pooled estimates from all imputations are reported. For the SLEDAI analysis, we included baseline SLEDAI score as we hypothesised that the impact of this variable should be substantial, and removing it could therefore be misleading by making relatively less impactful variables appear to have greater influence.

Sensitivity analysis compared trajectories that used SLEDAI 2K scores with complete versus incomplete data for urinary variables. As with the original analysis, the trajectories were assessed based on fit statistics and clinical relevance.

## Results

### Baseline characteristics

**Figure 1:**
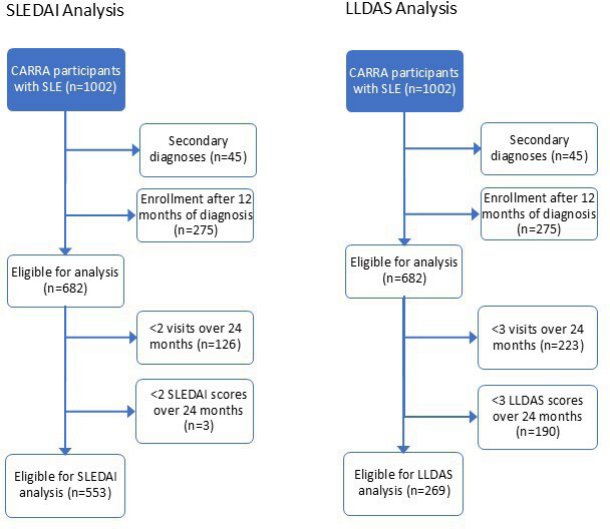
CONSORT diagram of selection of patient populations for the SLEDAI 2K and LLDAS analyses. CARRA, Childhood Arthritis and Rheumatology Research Alliance; CONSORT, Consolidated Standards of Reporting Trials; LLDAS, lupus low disease activity state; SLEDAI 2K, SLE Disease Activity Index 2000.

Of 1002 patients with pSLE identified in the CARRA Registry, 553 met criteria for the SLEDAI 2K analysis and 269 for the LLDAS analyses (figure 1, CONSORT (Consolidated Standards of Reporting Trials) diagram). The mean and median number of visits was 3.9 and 4.0 for the SLEDAI 2K analysis, and 4.7 and 5.0 for the LLDAS analysis, respectively.

In the SLEDAI 2K analysis, 87% were female, with a median age of 14 years at diagnosis (IQR 12–16) (table 1). Approximately one-quarter each identified as black/African American/African/Afro-Caribbean, Hispanic/Latino/Spanish and white. Among parents, 44% reported at least some college education and an additional 17% some graduate school. Annual household income was <US$50 000 for 48% and ≥US$100 000 for 25%. Only 3% of patients were uninsured. Median rank ADI was 47 (IQR 23–73). Lupus disease burden was significant, with median ACR classification criteria score of 5 (IQR 4–6) and median baseline SLEDAI 2K of 8 (IQR 3–14). Lupus nephritis was present in 38% of patients (199/527). For treatment at or before baseline, most (87%) were on hydroxychloroquine, with stronger immunosuppressive medications including mycophenolate mofetil (40%), rituximab (11%), cyclophosphamide (11%), intravenous pulse dose steroids (29%) and high-dose oral steroids (10%). In regard to PRO collection, 80% (441/553) of patients with data on pain intensity reported pain, with a median pain intensity score of 2 (IQR 0–4) and median pain interference T score of 50 (IQR 34–60).

**Table 1:** Baseline demographic and clinical characteristics by SLEDAI 2K trajectories

	Total sample (n=553)	Trajectory 1 Low+stable (n=421)	Trajectory 2 High+decreasing (n=71)	Trajectory 3 Intermediate+stable (n=61)	P value
Sociodemographic characteristics					
Age at symptom onset (years), median (IQR)	13 (11–15)	13 (11–15)	14 (12–16)	14 (11–15)	0.47
Age at diagnosis (years), median (IQR)	14 (12–16)	14 (12–16)	14 (12–16)	14 (12–16)	0.87
Female sex	481/553 (87%)	362/421 (86%)	63/71 (89%)	56/61 (92%)	0.40
Race/ethnicity					0.05
Asian	60/541 (11%)	49/409 (12%)	8/71 (11%)	3/61 (5%)	
Black, African American, African or Afro-Caribbean	136/541 (25%)	98/409 (24%)	12/71 (17%)	26/61 (42%)	
Hispanic, Latino or Spanish origin	130/541 (24%)	96/409 (24%)	19/71 (27%)	15/61 (25%)	
White	136/541 (25%)	103/409 (25%)	21/71 (30%)	12/61 (20%)	
Other/multiple races	79/541 (15%)	63/409 (15%)	11/71 (16%)	5/61 (8%)	
Insurance					0.001
Private	241/552 (44%)	189/420 (45%)	32/71 (45%)	20/61 (33%)	
Government*	230/552 (42%)	172/420 (41%)	27/71 (38%)	31/61 (51%)	
Other	64/552 (12%)	53/420 (13%)	5/71 (7%)	6/61 (10%)	
None	17/552 (3%)	6/420 (1%)	7/71 (10%)	4/61 (7%)	
Highest level of parent/guardian education†					0.21
Less than high school	56/465 (12%)	40/353 (11%)	10/64 (16%)	6/48 (13%)	
Graduated high school	127/465 (27%)	96/353 (27%)	17/64 (27%)	14/48 (29%)	
College (between 1—4 years)	203/465 (44%)	149/353 (42%)	48/64 (44%)	26/48 (54%)	
Graduate school	79/465 (17%)	68/353 (19%)	9/64 (14%)	2/48 (4%)	
Household income (annual gross) in US$†					0.48
<US$25 000	71/331 (21%)	52/246 (21%)	11/48 (23%)	8/37 (22%)	
US$25 000—49 999	87/331 (26%)	66/246 (27%)	11/48 (23%)	10/37 (27%)	
US$50 000—74 999	49/331 (15%)	32/246 (13%)	7/48 (15%)	10/37 (27%)	
US$75 000—99 999	41/331 (12%)	31/246 (13%)	6/48 (13%)	4/37 (11%)	
US$100 000—150 000	36/331 (11%)	31/246 (13%)	5/48 (10%)	0/37 (0%)	
Above US$150 000	47/331 (14%)	34/246 (14%)	8/48 (17%)	5/37 (14%)	
Area Deprivation Index, national percentile, median (IQR)†	47 (23–73)	44 (21–72)	52 (32–78)	64 (33–76)	0.03
Indices and classification criteria at baseline Registry visit					
Days from onset to diagnosis, median (IQR)	81 (33–193)	83 (34–203)	46 (25–134)	88 (33–217)	0.03
Days from diagnosis to baseline, median (IQR)	63 (19–160)	78 (31–185)	15 (7–39)	42 (13–137)	<0.001
ACR classification criteria score, median (IQR)	5 (4–6)	4 (4–5)	6 (5–7)	5 (4–6)	<0.001
SLICC classification criteria score, median (IQR)	9 (7–10)	8 (6–10)	11 (9–13)	9 (8–11)	<0.001
SLEDAI 2K score—baseline window, median (IQR)	8 (3–14)	5 (3–10)	24 (21–27)	13 (9–16)	<0.001
SLICC/ACR Damage Index score, median (IQR)	0 (0–0)	0 (0–0)	0 (0–1)	0 (0–1)	<0.001
Physician global assessment, median (IQR)†	3 (1–4)	2 (1–4)	5 (3–6)	4 (2–6)	<0.001
Patient/parent global assessment, median (IQR)†	2 (0–5)	2 (0–5)	3 (1–5)	2 (0–5)	0.02
Paediatric global health survey score, T score†	39 (34–42)	39 (34–42)	36 (32–40)	39 (36–46)	0.004
Pain intensity score, median (IQR)†	2 (0–4)	2 (0–4)	4 (2–6)	2 (0–5)	<0.001
Pain interference score, T score, median (IQR)†	50 (34–60)	47 (34–59)	58 (51–62)	51 (34–62)	<0.001
Lupus nephritis	199/527 (38%)	132/403 (33%)	39/67 (58%)	28/57 (49%)	<0.001
Medication Use					
Days from symptom onset to first immunosuppression, median (25th, 75th)†	96 (40, 228)	101 (46, 243)	67 (27, 152)	104 (50, 273)	0.009
Oral prednisone use					0.33
None	448/553 (88%)	367/421 (87%)	67/71 (94%)	54/61 (89%)	
Low to intermediate dose	11/553 (2%)	10/421 (2%)	1/71 (1%)	0/61 (0%)	
High dose‡	54/553 (10%)	44/421 (10%)	3/71 (4%)	7/61 (11%)	
Intravenous pulse steroids	159/553 (29%)	111/421 (26%)	29/71 (41%)	19/61 (31%)	0.04
Cyclophosphamide	59/553 (11%)	43/421 (10%)	8/71 (11%)	8/61 (13%)	0.78
Rituximab	60/553 (11%)	46/421 (11%)	9/71 (13%)	5/61 (8%)	0.71
Mycophenolate mofetil	220/553 (40%)	160/421 (38%)	32/71 (45%)	28/61 (46%)	0.31
Azathioprine	64/553 (12%)	53/421 (13%)	4/71 (6%)	7/61 (12%)	0.24
Methotrexate	54/553 (10%)	45/421 (11%)	4/71 (6%)	5/61 (8%)	0.38
Hydroxychloroquine	480/553 (87%)	373/421 (89%)	53/71 (75%)	54/61 (89%)	0.005

* Includes Medicare, Medicaid, state and military insurances.

† Variables with missingness >5%.

‡ High dose oral prednisone defined as >20 mg/day.

ACR, American College of Rheumatology; SLEDAI 2K, SLE Disease Activity Index 2000; SLICC, Systemic Lupus International Collaborating Clinics.

When comparing participants who were included (n=553) versus excluded (n=449) in the SLEDAI 2K analysis, those included had an older average age at symptom onset (mean 13.0 years (SD 2.9) vs 12.6 (SD 3.1), p=0.02); higher national ADI (mean 48.4 (SD 28.7) vs 44.1 (SD 28.2), p=0.04); differences in parent/guardian education; higher average physician global score (mean 2.9 (SD 2.2) vs 2.1 (SD 2.2), p<0.0001), baseline SLEDAI score (mean 9.7 (SD 8.1) vs 7.0 (SD 6.6), p<0.0001), pain interference T score (mean 49.0 (SD 12.4) vs 47.2 (SD 12.0), p=0.04); more oral steroid use (65 (11.8%) vs 33 (7.3%), p=0.02); and less use of certain medications including methotrexate (54 (9.8%) vs 70 (15.6%), p=0.005) and mycophenolate mofetil (220 (39.8%) vs 217 (48.3%), p=0007) (online supplemental table 2).

In the LLDAS cohort, those included (n=269) compared with those excluded (n=733) had higher average physician global scores (mean 3.0 (SD 2.2) vs 2.4 (SD 2.2), p<0.0001), baseline SLEDAI scores (mean 9.9 (SD 7.8) vs 8.1 (SD 7.5), p=0.0004), SLICC classification criteria scores (mean 8.8 (SD 2.5) vs 8.4 (SD 2.9), p=0.04) and ACR classification criteria scores (mean 4.9 (SD 1.5) vs 4.7 (SD 1.6), p=0.03); and lower use of mycophenolate mofetil (101 (38%) vs 336 (46%), p=0.02) and rituximab (22 (8%) vs 95 (13%), p=0.04) (online supplemental table 3).

### SLEDAI 2K trajectories

**Figure 2:**
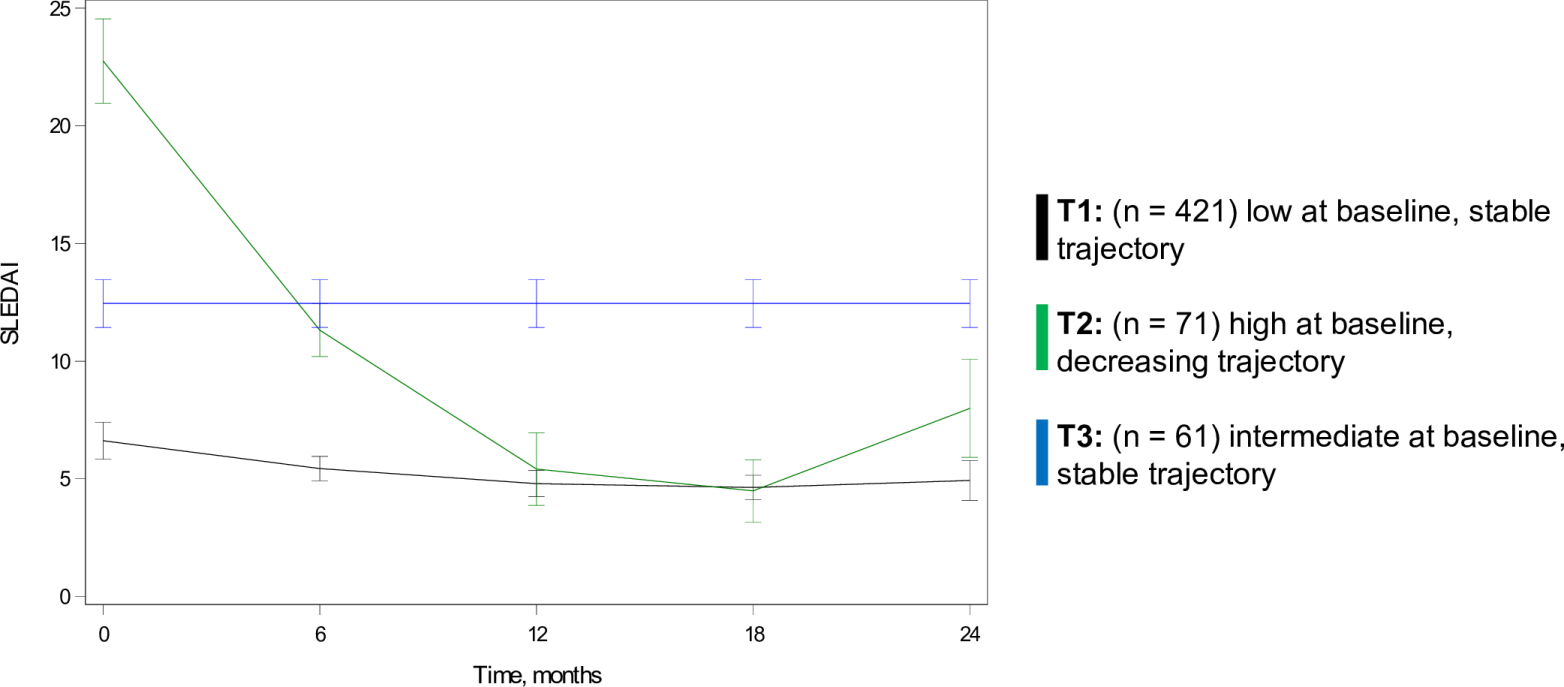
Latent trajectory analysis of SLEDAI 2K scores over 24 months in the CARRA Lupus Registry. CARRA, Childhood Arthritis and Rheumatology Research Alliance; SLEDAI 2K, SLE Disease Activity Index 2000.

The BIC, AIC and average posterior probabilities are presented for the top model of SLEDAI 2K trajectories (online supplemental table) and shown over time (figure 2). Trajectories of SLEDAI 2K scores included (T1) low and stable (n=421, 76%), (T2) high and decreasing (n=71, 13%), (T3) intermediate and stable (n=61, 11%).

### The impact of covariates on SLEDAI 2K trajectories

Baseline characteristics significantly differed between SLEDAI 2K trajectories in several categories (table 1).

In multinomial generalised logistic regression, including the same variables as those described in table 1, only baseline SLEDAI 2K score and insurance type remained statistically significant (table 2). Specifically, SLEDAI 2K scores (per 1-point increase) were higher in T2 and T3 versus T1, and compared with private insurance, government insurance was less common in T2 versus T1 and no insurance was more common in T3 versus T1.

**Table 2:** Significant associations in logistic regression models for baseline characteristics with SLEDAI 2K trajectories

Comparison	OR (95% CI)*†	P value
SLEDAI 2K trajectories‡		
Baseline SLEDAI 2K score (per 1 point increase)		
Trajectory 1	Reference	
Trajectory 2	2.37 (1.88 to 2.99)	<0.001
Trajectory 3	1.23 (1.16 to 1.30)	<0.001
Insurance		
Government versus private insurance		
Trajectory 1	Reference	
Trajectory 2	0.13 (0.04 to 0.45)	0.001
Trajectory 3	0.77 (0.45 to 1.34)	0.36
No insurance versus private insurance		
Trajectory 1	Reference	
Trajectory 2	4.44 (0.58 to 34.25)	0.15
Trajectory 3	3.83 (1.24 to 11.82)	0.02
Other insurance versus private insurance		
Trajectory 1	Reference	
Trajectory 2	1.93 (0.45 to 8.23)	0.37
Trajectory 3	0.65 (0.30 to 1.41)	0.27

* Multinomial generalised logistic regression with forward variable selection. Variables must have p value of ≤0.05 to stay in the model.

† Variables considered for inclusion in the model are age at symptom onset, days from onset to diagnosis, days from diagnosis to baseline, race, parental education level, parental income, insurance, medication (cyclophosphamide, methotrexate, mycophenolate mofetil, azathioprine, hydroxychloroquine, rituximab, days from onset to immunosuppressant, ACE inhibitor/angiotensin receptor blocker), oral dose steroid use, intravenous pulse steroid use, classification of days to cyclophosphamide use, lupus nephritis, baseline SLEDAI 2K score, Systemic Lupus International Collaborating Clinics (SLICC) Damage Index score, American College of Rheumatology classification criteria score, SLICC classification criteria score, pain intensity score, pain interference score, physician global assessment, patient/parent global assessment and Area Deprivation Index national score.

‡ Multinomial generalised logistic regression with forward variable selection. Trajectories defined as trajectory 1, low and stable; trajectory 2, high and decreasing; and trajectory 3, intermediate and stable.

SLEDAI 2K, SLE Disease Activity Index 2000.

### Supplemental sensitivity analysis for SLEDAI 2K urinary variables

Missing data for urinary variables in the SLEDAI 2K was higher than for other SLEDAI 2K variables. We performed a supplemental sensitivity analysis comparing trajectories using a SLEDAI 2K that had complete urinary data versus incomplete data, and the same trajectories were selected with fewer numbers in each group (data not shown).

### First LLDAS assessment and LLDAS attainment over the study period

17% (47/269) of patients attained LLDAS at first assessment; the first assessment was at the 6-month visit for most participants (89%). Most participants (85%) also had a 24-month visit. By 24 months, LLDAS attainment at any point in the study period increased to 51% (136/269). When comparing baseline characteristics between participants who had not achieved LLDAS at first assessment versus those who had, participants who had higher baseline SLEDAI 2K, SDI and ACR classification criteria as well as worse scores on PROs (pain intensity, pain interference and paediatric global health survey) were less likely to attain LLDAS at first assessment (table 3). In logistic regression, differences in baseline SDI and pain interference score remained statistically significant, with higher SDI and higher pain interference scores associated with lower odds of LLDAS achievement at first assessment.

**Table 3:** Significant baseline demographic and clinical characteristics of patients attaining LLDAS at first follow-up visit with logistic regression modelling

	First assessed LLDAS		P value
	No (n=222)	Yes (n=47)	
ACR classification criteria score, median (IQR)	5 (4–6)	5 (3–5)	0.04
SLEDAI 2K score—baseline window, median (IQR)	9 (4–15)	5 (3–11)	0.01
SLICC Damage Index score, median (IQR)	0 (0–1)	0 (0–0)	0.008
Pain intensity score, median (IQR)	2 (0–5)	1 (0–3)	0.05
Pain interference score, T score, median (IQR)	51.7 (34.0–59.5)	40.6 (34.0–49.5)	0.002

Logistic regression model for LLDAS attainment at first follow-up visit	OR (95% CI)*†	P value
SLICC Damage Index score (per 1 point increase)	0.34 (0.13 to 0.90)	0.03
Pain interference score (per 1 point increase)	0.94 (0.90 to 0.99)	0.01

* Multinomial generalised logistic regression with forward variable selection. Variables must have a p value of ≤0.05 to stay in the model.

† Variables considered for inclusion in the model are age at symptom onset, days from onset to diagnosis, days from diagnosis to baseline, race, parental education level, parental income, insurance, medication (cyclophosphamide, methotrexate, mycophenolate mofetil, azathioprine, hydroxychloroquine, rituximab, days from onset to immunosuppressant, ACE inhibitor/angiotensin receptor blocker), oral dose steroid use, intravenous pulse steroid use, classification of days to cyclophosphamide use, lupus nephritis, baseline SLEDAI 2K score, SLICC Damage Index score, ACR classification criteria score, SLICC classification criteria score, pain intensity score, pain interference score, physician global assessment, patient/parent global assessment and Area Deprivation Index national score.

ACR, American College of Rheumatology; LLDAS, lupus low disease activity state; SLEDAI 2K, SLE Disease Activity Index 2000; SLICC, Systemic Lupus International Collaborating Clinics.

The relationships between baseline characteristics and achieving LLDAS during the 24 months following enrolment were also assessed (table 4), with parent/guardian education, SLEDAI 2K, ACR and SLICC classification criteria, pain interference and patient/parent global assessment all predictive of LLDAS attainment over the study period in bivariate analysis. Highest level of parent/guardian education completed, rituximab use at baseline, baseline ACR classification criteria and pain interference remained significant in logistic regression. Specifically, for patients who achieved LLDAS within 24 months, baseline rituximab use was higher, the number of baseline ACR classification criteria was lower and pain interference scores were lower compared with those who did not achieve LLDAS. Regarding parent/guardian education, patients with a parent/guardian who completed graduate school had higher odds of achieving LLDAS compared with patients with a parent/guardian who completed some or graduated college as well as with those who graduated high school.

**Table 4:** Baseline demographic and clinical characteristics of patients attaining LLDAS within 24 months of enrolment with logistic regression modelling

	Achieved LLDAS		P value
	No (n=133)	Yes (n=136)	
Highest level of parent/guardian education completed			<0.001
Less than high school graduate	14/113 (12%)	17/119 (14%)	
Graduated high school	33/113 (29%)	28/119 (24%)	
College	61/113 (54%)	47/119 (40%)	
Graduate school	5/113 (4%)	27/119 (23%)	
ACR classification criteria score, median (IQR)	5 (4–6)	5 (4–6)	0.02
SLICC classification criteria score, median (IQR)	9 (7–11)	8 (7–10)	0.02
SLEDAI 2K score—baseline window, median (IQR)	10 (4–16)	7 (3–13)	0.004
Physician global assessment, median (IQR)	3 (1–5)	2 (1–4)	0.008
Patient/parent global assessment, median (IQR)	2 (0–5)	1 (0–4)	0.04
Pain interference score, T score, median (IQR)	53 (34–62)	45 (34–58)	0.007

Logistic regression model for LLDAS attainment within 24 months	OR (95% CI)*†	P value
Highest level of parent/guardian education		
Graduate school	Reference	
Graduated high school	0.59 (0.37 to 0.95)	0.03
College (1–4 year college, junior college or technical school)	0.56 (0.37 to 0.85)	0.006
<High school	1.02 (0.56 to 1.89)	0.94
Rituximab	2.73 (1.03 to 7.20)	0.04
ACR classification criteria score	0.78 (0.65 to 0.94)	0.008
Pain interference score	0.97 (0.95 to 0.99)	0.01

* Multinomial generalised logistic regression with forward variable selection. Variables must have p value of ≤0.10 to stay in the model.

† Variables considered for inclusion in the model are age at symptom onset, days from onset to diagnosis, days from diagnosis to baseline, race, parental education level, parental income, insurance, medication (cyclophosphamide, methotrexate, mycophenolate mofetil, azathioprine, hydroxychloroquine, rituximab, days from onset to immunosuppressant, ACE inhibitor/angiotensin receptor blocker), oral dose steroid use, intravenous pulse steroid use, classification of days to cyclophosphamide use, lupus nephritis, baseline SLEDAI 2K score, SLICC Damage Index score, ACR classification criteria score, SLICC classification criteria score, pain intensity score, pain interference score, physician global assessment, patient/parent global assessment and Area Deprivation Index national score.

ACR, American College of Rheumatology; LLDAS, lupus low disease activity state; SLEDAI 2K, SLE Disease Activity Index 2000; SLICC, Systemic Lupus International Collaborating Clinics.

## Discussion

This study was designed to identify predictors of disease activity trajectories and LLDAS attainment in newly diagnosed pSLE participants in the CARRA Registry. In this diverse cohort of patients with active pSLE, we identified three distinct disease activity trajectories that were predicted by baseline SLEDAI 2K scores and health insurance, with lack of insurance associated with higher disease activity over time and government insurance less associated with high disease activity at enrolment. Furthermore, higher levels of pain interference were related to lower rates of LLDAS achievement, both at first assessment and over the study period of 24 months.

SLEDAI 2K trajectories for pSLE have been described in a Canadian cohort.^6^ The Canadian study had a longer median follow-up time of 5.4 years and identified five disease activity trajectories for SLEDAI 2K and prednisone exposure, specifically (1) relapsing/transforming; (2) high initial activity with long-term remission; (3) moderate initial activity with long-term, low-grade disease; (4) late relapsing; and (5) chronic, low-grade.^6^ While the shorter time frame of our study makes direct comparison difficult, the studies are similar in identifying trajectories with ongoing low-grade disease. Identification of low-grade disease activity is important in therapeutic management, since even low levels of disease activity as measured by SLEDAI 2K scores can lead to increased damage.^19^ In fact, disease activity trajectories in the Canadian study were associated with disease damage trajectories.^6^

In our cohort, 17% of patients attained LLDAS at the first assessment (second Registry visit for most included participants) and achieving LLDAS at any point in the study period increased to 51% by 24 months. Achievement of LLDAS at the first or subsequent assessments was predicted by fewer ACR classification criteria at enrolment and lower baseline pain interference; subsequent LLDAS attainment was also predicted by higher parent/guardian education level and baseline rituximab use. In a pSLE cohort in the UK assessing LLDAS, 67% achieved LLDAS over a median of 18 months (IQR 8.5—30.8).^20^ Achievement of LLDAS in that study was associated with a significantly lower risk of severe flare and less damage over time.^20^ It is possible that rates of LLDAS attainment differed between the UK cohort and ours due to the number of visits per participant; their participants had a median of 10 visits over 2 years, allowing for more frequent assessment of LLDAS.

The importance of pain-related PROs was underscored by their association with LLDAS achievement as well as the high amount (64%) of patients who reported some degree of pain at baseline. Pain in pSLE is described as an important factor in health-related quality of life (HRQoL)^21^ and disability,^22^ but its relationship to disease activity is less well described. Interestingly, a previous study did not find a relationship between more detailed PROMIS paediatric measures including pain interference and dichotomised SLEDAI score of active versus inactive disease, potentially reflecting the small sample size.^8^ In another CARRA Registry study of adolescents with pSLE, patients reporting higher levels of pain were more likely to have discordance with their provider in rating their health status.^23^ This discordance in health status ratings may result in provider undertreatment and therefore worse disease activity. In adult studies, PROs and HRQoL have not been associated with adjusted mean SLEDAI-2K in joint trajectory modelling.^24^ However, one study in adults with SLE described trajectories based on a combination of disease activity measures and PROs,^25^ and there are data suggesting that higher self-reported pain in adult patients with lupus is associated with higher SLEDAI scores.^26^ Further work should explore what aspects of pain are most impactful on function and the relationship of longitudinal PROs and disease activity measures over time.

This study demonstrated an important association between lacking health insurance and having a trajectory of higher disease activity in a pSLE population. This does not appear to reflect a limited ability to attend follow-up visits, as the median number of follow-up visits was similar across the trajectories. Other factors, such as inability to afford medications or diagnostic testing, may contribute. Interestingly, national ADI was likewise associated with higher disease activity trajectories in the univariate, but not the multivariate analysis, while household income and parental/guardian education levels were similar across trajectories. Prior studies have shown significant disparities in disease damage accrual by SES and race, with one study in adults showing that SES variables accounted for 10% of organ damage measured by the SDI.^27^

We also found a relationship between LLDAS and parental/guardian education, with LLDAS achievement being more likely for patients with parents/guardians who attended graduate school compared with those graduating high school or attending college. This could potentially reflect many factors, such as higher health literacy or greater flexibility at the parent/guardian’s workplace to accommodate medical care. The relationships between SLEDAI trajectories and LLDAS achievement with being uninsured and parent/guardian education warrant further investigation to identify modifiable factors.

The only medication in our study that was associated with a disease activity measure (LLDAS) was rituximab use at baseline. It is possible that rituximab is more effective at controlling early disease activity over the first 2 years of enrolment as described in our study. Otherwise, short-term disease activity was not predicted by baseline medication use.

Limitations of this study include missing data, with the highest percentages of missing data in household income, ADI, PROs on health status and days from diagnosis to immunosuppression (table 1). Our analysis used multiple imputation for missing data. Some data collection was done during the COVID-19 pandemic which impacted missingness, including PROs, and may also have limited accuracy of physician-reported outcomes in telemedicine visits. Additionally, predictors of SLEDAI 2K trajectories were based on SLEDAI 2K scores calculated at 6-month windows per CARRA Registry procedures for data entry. In the trajectory analysis, the small mean change in the groups over time was based on the average score of all patients in the group; individual patients within the group may still have experienced flares. Also, our inclusion criteria may have selected for a sicker population of patients with pSLE, given that patients included in the SLEDAI 2K analysis had higher baseline SLEDAI 2K scores than those who were excluded.

Future directions include exploring these trajectories over a longer period of time, examining disease activity measures that are specific to pSLE,^28^ and whether identification of predictors of disease trajectories justifies tailoring immunosuppressive treatment at baseline. The role of pain interference in pSLE warrants greater attention, given its ability to predict lack of LLDAS attainment over the short and longer term. Additionally, patients with pSLE with high SLEDAI 2K scores at baseline should be closely monitored for persistent or worsening disease activity given the known relationship between disease activity and damage,^20^ and efforts should be made to identify and assist patients who lack health insurance.

## Supplementary material

10.1136/lupus-2025-001521 online supplemental file 1

## Data Availability

Data are available upon reasonable request.
