# Supplementary material for: Disease activity trajectories in paediatric lupus and associations with socioeconomic factors and patient-reported pain
Source: Lupus Sci Med. 2025 Aug 14;12(2):e001521. doi: 10.1136/lupus-2025-001521 (PMC12352166; doi:10.1136/lupus-2025-001521)
Supplement: online supplemental file 1 [file lupus-12-2-s001.docx]

**Supplemental table:**

**Supplemental table 1**. **Model fit statistics for top ten SLEDAI 2K latent trajectory models**

| BIC | AIC | Trajectory | n | Posterior Probability |
| --- | --- | --- | --- | --- |
| -6643.85 | -6622.27 | 1 | 421 | 0.94 |
|  |  | 2 | 71 | 0.85 |
|  |  | 3 | 61 | 0.82 |
| -6646.37 | -6622.64 | 1 | 53 | 0.81 |
|  |  | 2 | 419 | 0.95 |
|  |  | 3 | 81 | 0.84 |
|  |  |  |  |  |
| -6646.61 | -6627.19 | 1 | 414 | 0.94 |
|  |  | 2 | 62 | 0.80 |
|  |  | 3 | 77 | 0.85 |
|  |  |  |  |  |
| -6648.77 | -6627.19 | 1 | 415 | 0.94 |
|  |  | 2 | 50 | 0.80 |
|  |  | 3 | 88 | 0.85 |
|  |  |  |  |  |
| -6648.81 | -6622.91 | 1 | 420 | 0.95 |
|  |  | 2 | 50 | 0.81 |
|  |  | 3 | 83 | 0.85 |
|  |  |  |  |  |
| -6655.49 | -6638.23 | 1 | 93 | 0.84 |
|  |  | 2 | 400 | 0.93 |
|  |  | 3 | 60 | 0.79 |
|  |  |  |  |  |
| -6657.53 | -6638.11 | 1 | 109 | 0.84 |
|  |  | 2 | 401 | 0.93 |
|  |  | 3 | 43 | 0.83 |
|  |  |  |  |  |
| -6704.11 | -6686.85 | 1 | 435 | 0.94 |
|  |  | 2 | 118 | 0.87 |
|  |  |  |  |  |
| -6705.46 | -6690.36 | 1 | 425 | 0.94 |
|  |  | 2 | 128 | 0.86 |
|  |  |  |  |  |
| -6706.40 | -6684.82 | 1 | 25 | 0.83 |
|  |  | 2 | 434 | 0.95 |
|  |  | 3 | 94 | 0.79 |

Abbreviations: ACI = Akaike Information Criterion; BIC = Bayesian Information Criterion; SLEDAI 2K = Systemic Lupus Erythematosus Disease Activity Index 2000.

**Supplemental table 2. Baseline demographic and clinical characteristics in the SLEDAI 2K analysis**

|  | **Total Sample** (n=1002) | **Excluded**  (n = 449) | **Included**  (n = 553) | ***P* value** |
| --- | --- | --- | --- | --- |
| **Sociodemographic characteristics** | | | | |
| Age at symptom onset (years), median (IQR) | 13 (11, 15) | 13 (11, 15) | 13 (11, 15) | 0.02 |
| Age at diagnosis (years), median (IQR) | 14 (12, 16) | 14 (11, 16) | 14 (12, 16) | 0.06 |
| Female sex | 868/1002 (87%) | 387/449 (86%) | 481/553 (87%) | 0.72 |
| Race/ethnicity |  |  |  | 0.11 |
| Asian | 126/972 (13%) | 66/431 (15%) | 60/541 (11%) |  |
| Black, African American,  African, or Afro-Caribbean | 260/972 (27%) | 124/431 (29%) | 136/541 (25%) |  |
| Hispanic, Latino, or Spanish origin | 218/972 (22%) | 88/431 (20%) | 130/541 (24%) |  |
| White | 239/972 (25%) | 103/431 (24%) | 136/541 (25%) |  |
| Other/multiple races | 129/972 (13%) | 50/431 (12%) | 79/541 (15%) |  |
| Insurance |  |  |  | 0.11 |
| Private | 440/996 (44%) | 199/444 (45%) | 241/552 (44%) |  |
| Government* | 423/996 (42%) | 193/444 (43%) | 230/552 (42%) |  |
| Other | 112/996 (11%) | 48/444 (11%) | 64/552 (12%) |  |
| None | 21/996 (2%) | 4/444 (1%) | 17/552 (3%) |  |
| Highest level of parent/guardian education† |  |  |  | 0.03 |
| Less than high school | 93/806 (12%) | 37/341 (11%) | 56/465 (12%) |  |
| Graduated high school | 196/806 (24%) | 69/341 (20%) | 127/465 (27%) |  |
| College (between 1—4 years) | 358/806 (44%) | 155/341 (45%) | 203/465 (44%) |  |
| Graduate school | 159/806 (20%) | 80/341 (23%) | 79/465 (17%) |  |
| Household income (annual gross) in USD † |  |  |  | 0.35 |
| <$25,000 | 112/573 (20%) | 41/242 (17%) | 71/331 (21%) |  |
| $25,000—$49,999 | 151/573 (26%) | 64/242 (26%) | 87/331 (26%) |  |
| $50,000—$74,999 | 77/573 (13%) | 28/242 (12%) | 49/331 (15%) |  |
| $75,000—$99,999 | 72/573 (13%) | 31/242 (13%) | 41/331 (12%) |  |
| $100,000—$150,000 | 74/573 (13%) | 38/242 (16%) | 36/331 (11%) |  |
| Above $150,000 | 87/573 (15%) | 40/242 (17%) | 47/331 (14%) |  |
| Area Deprivation Index, national percentile, median (IQR)† | 45 (21, 71) | 42 (19, 65) | 47 (23, 73) | 0.04 |
| **Indices and Classification Criteria at Baseline Registry Visit** | | | | |
| Days from diagnosis to baseline, median (IQR) | 140 (38, 420) | 466 (161, 711) | 63 (19, 160) | <0.001 |
| ACR classification criteria score, median (IQR) | 5 (4, 6) | 5 (4, 6) | 5 (4, 6) | 0.26 |
| SLICC classification criteria score, median (IQR) | 8 (6, 10) | 8 (6, 10) | 9 (7, 10) | 0.15 |
| SLEDAI 2K score – baseline window, median (IQR) | 7 (3, 13) | 5 (3, 10) | 8 (3, 14) | <0.001 |
| SLICC/ACR Damage Index score, median (IQR) | 0 (0, 0) | 0 (0, 0) | 0 (0, 0) | 0.26 |
| Physician global assessment, median (IQR) † | 2 (1, 4) | 2 (1, 3) | 3 (1, 4) | <0.001 |
| Patient/parent global assessment, median (IQR) † | 2 (0, 4) | 2 (0, 4) | 2 (0, 5) | 0.06 |
| Pediatric global health survey score, T score † | 39 (34, 42) | 39 (34, 42) | 39 (34, 42) | 1.0 |
| Pain intensity score, median (IQR) † | 2 (0, 4) | 1 (0, 4) | 2 (0, 4) | 0.10 |
| Pain interference score, T score, median (IQR) † | 48 (34, 59) | 47 (3, 57) | 50 (34, 60) | 0.04 |
| Lupus nephritis | 363/932 (39%) | 164/405 (40%) | 199/527 (38%) | 0.40 |
| **Medication Use** | | | | |
| Days from symptom onset to 1^st^ immunosuppression, median (25^th^, 75^th^) † | 15 (1, 92) | 22 (2, 129) | 6 (0, 28) | <0.001 |
| Oral prednisone use |  |  |  | 0.02 |
| None | 904/1002 (90%) | 416/449 (93%) | 488/553 (88%) |  |
| Low to intermediate dose | 21/1002 (2%) | 10/449 (2%) | 11/553 (2%) |  |
| High dose‡ | 77/1002 (8%) | 23/449 (5%) | 54/553 (10%) |  |
| Cyclophosphamide | 105/1002 (10%) | 46/449 (10%) | 59/553 (11%) | 0.83 |
| Rituximab | 117/1002 (12%) | 57/449 (13%) | 60/553 (11%) | 0.37 |
| Mycophenolate mofetil | 437/1002 (44%) | 217/449 (48%) | 220/553 (40%) | 0.007 |
| Azathioprine | 117/1002 (12%) | 53/449 (12%) | 64/553 (12%) | 0.91 |
| Methotrexate | 124/1002 (12%) | 70/449 (16%) | 54/553 (10%) | 0.005 |
| Hydroxychloroquine | 870/1002 (87%) | 390/449 (87%) | 480/553 (87%) | 0.98 |

Abbreviations: ACR = American College of Rheumatology; IQR = interquartile range; SLEDAI 2K = Systemic Lupus Erythematosus Disease Activity Index 2000; SLICC = Systemic Lupus International Collaborating Clinics

* Includes Medicare, Medicaid, State and Military insurances

† Variables with missingness >5%

‡ High dose oral prednisone defined as >20mg/day

**Supplemental table 3. Baseline demographic and clinical characteristics in the LLDAS analysis**

|  | **Total Sample** (n=1002) | **Excluded**  (n = 733) | **Included**  (n = 269) | ***P* value** |
| --- | --- | --- | --- | --- |
| **Sociodemographic characteristics** | | | | |
| Age at symptom onset (years), median (IQR) | 13 (11, 15) | 13 (11, 15) | 13 (11, 15) | 0.49 |
| Age at diagnosis (years), median (IQR) | 14 (12, 16) | 14 (12, 16) | 14 (11, 16) | 0.92 |
| Female sex | 868/1002 (87%) | 637/733 (87%) | 231/269 (86%) | 0.67 |
| Race/ethnicity |  |  |  | 0.81 |
| Asian | 126/972 (13%) | 94/709 (13%) | 32/263 (12%) |  |
| Black, African American,  African, or Afro-Caribbean | 260/972 (27%) | 192/709 (27%) | 68/263 (26%) |  |
| Hispanic, Latino, or Spanish origin | 218/972 (22%) | 152/709 (21%) | 66/263 (25%) |  |
| White | 239/972 (25%) | 175/709 (25%) | 64/263 (24%) |  |
| Other/multiple races | 129/972 (13%) | 96/709 (14%) | 33/263 (13%) |  |
| Insurance |  |  |  | 0.97 |
| Private | 440/996 (44%) | 319/727 (44%) | 121/269 (45%) |  |
| Government* | 423/996 (42%) | 312/727 (43%) | 111/269 (41%) |  |
| Other | 112/996 (11%) | 81/727 (11%) | 31/269 (12%) |  |
| None | 21/996 (2%) | 15/727 (2%) | 6/269 (2%) |  |
| Highest level of parent/guardian education† |  |  |  | 0.05 |
| Less than high school | 93/806 (12%) | 62/574 (11%) | 31/232 (13%) |  |
| Graduated high school | 196/806 (24%) | 135/574 (24%) | 61/232 (26%) |  |
| College (between 1—4 years) | 358/806 (44%) | 250/574 (44%) | 108/232 (47%) |  |
| Graduate school | 159/806 (20%) | 127/574 (22%) | 32/232 (14%) |  |
| Household income (annual gross) in USD † |  |  |  | 0.85 |
| <$25,000 | 112/573 (20%) | 77/407 (19%) | 35/166 (21%) |  |
| $25,000—$49,999 | 151/573 (26%) | 106/407 (26%) | 45/166 (27%) |  |
| $50,000—$74,999 | 77/573 (13%) | 54/407 (13%) | 23/166 (14%) |  |
| $75,000—$99,999 | 72/573 (13%) | 50/407 (12%) | 22/166 (13%) |  |
| $100,000—$150,000 | 74/573 (13%) | 53/407 (13%) | 21/166 (13%) |  |
| Above $150,000 | 87/573 (15%) | 67/407 (16%) | 20/166 (12%) |  |
| Area Deprivation Index, national percentile, median (IQR)† | 45 (21, 71) | 45 (21, 70) | 43 (23, 71) | 0.60 |
| **Indices and Classification Criteria at Baseline Registry Visit** | | | | |
| Days from diagnosis to baseline, median (IQR) | 140 (38, 420) | 216 (52, 548) | 62 (19, 161) | <0.001 |
| ACR classification criteria score, median (IQR) | 5 (4, 6) | 5 (4, 6) | 5 (4, 6) | 0.03 |
| SLICC classification criteria score, median (IQR) | 8 (6, 10) | 8 (6, 10) | 9 (7, 10) | 0.04 |
| SLEDAI 2K score – baseline window, median (IQR) | 7 (3, 13) | 5 (3, 11) | 9 (3, 15) | <0.001 |
| SLICC/ACR Damage Index score, median (IQR) | 0 (0, 0) | 0 (0, 0) | 0 (0, 0) | 0.30 |
| Physician global assessment, median (IQR) † | 2 (1, 4) | 2 (1, 4) | 3 (1, 5) | <0.001 |
| Patient/parent global assessment, median (IQR) † | 2 (0, 4) | 2 (0, 4) | 2 (0, 5) | 0.84 |
| Pediatric global health survey score, T score † | 39 (34, 42) | 37 (34, 42) | 39 (34, 44) | 0.14 |
| Pain intensity score, median (IQR) † | 2 (0, 4) | 2 (0, 4) | 2 (0, 4) | 0.47 |
| Pain interference score, T score, median (IQR) † | 48 (34, 59) | 48 (34, 58) | 50 (34, 60) | 0.51 |
| Lupus nephritis | 363/932 (39%) | 252/665 (38%) | 111/267 (42%) | 0.30 |
| **Medication Use** | | | | |
| Days from symptom onset to 1^st^ immunosuppression, median (25^th^, 75^th^) † | 15 (1, 92) | 22 (2, 129) | 6 (0, 28) | <0.001 |
| Oral prednisone use |  |  |  | 0.28 |
| None | 904/1002 (90%) | 668/733 (91%) | 236/269 (88%) |  |
| Low to intermediate dose | 21/1002 (2%) | 14/733 (2%) | 7/269 (3%) |  |
| High dose‡ | 77/1002 (8%) | 51/733 (7%) | 26/269 (10%) |  |
| Cyclophosphamide | 105/1002 (10%) | 69/733 (9%) | 36/269 (13%) | 0.07 |
| Rituximab | 117/1002 (12%) | 95/733 (13%) | 22/269 (8%) | 0.04 |
| Mycophenolate mofetil | 437/1002 (44%) | 336/733 (46%) | 101/269 (38%) | 0.02 |
| Azathioprine | 117/1002 (12%) | 84/733 (11%) | 33/269 (12%) | 0.72 |
| Methotrexate | 124/1002 (12%) | 45/733 (11%) | 4/269 (6%) | 0.38 |
| Hydroxychloroquine | 870/1002 (87%) | 633/733 (86%) | 237/269 (88%) | 0.47 |

Abbreviations: ACR = American College of Rheumatology; IQR = interquartile range; LLDAS: Lupus Low Disease Activity State; SLEDAI 2K = Systemic Lupus Erythematosus Disease Activity Index 2000; SLICC = Systemic Lupus International Collaborating Clinics

* Includes Medicare, Medicaid, State and Military insurances

† Variables with missingness >5%

‡ High dose oral prednisone defined as >20mg/day
